# A Parturient With Fontan Physiology and Suspected Placenta Accreta Spectrum: A Case Report

**DOI:** 10.7759/cureus.94494

**Published:** 2025-10-13

**Authors:** Taizoon Q Dhoon, Benjamin Rosellini, Michael Holland, Jean Vo, Yeyoon Choi, Shermeen Vakharia, Afshan Hameed, Elan Krojanker

**Affiliations:** 1 Anesthesiology, University of California, Irvine (UCI) Health, Orange, USA; 2 Anesthesiology and Perioperative Care, University of California, Irvine (UCI) Health, Orange, USA; 3 Anesthesiology and Perioperative Medicine, University of California, Irvine (UCI) Health, Orange, USA; 4 Obstetrics and Gynecology, University of California, Irvine (UCI) Health, Orange, USA

**Keywords:** breus' mole, congenital heart disease, ecmo, fontan physiology, hypoplastic left heart syndrome, massive subchorionic thrombohematoma, placenta accreta spectrum, pregnancy, single ventricle physiology, venous arterial extracorporeal membrane oxygenation

## Abstract

This manuscript reviews a complex case of a gravida 3, para 2 (G3P2) parturient with Fontan physiology, complicated by suspected placenta accreta spectrum (PAS) versus massive subchorionic thrombohematoma (MST), presenting with placental abruption at 32 weeks of gestation. This case highlights the intricacies of managing pregnancy in patients with single ventricle physiology, underscoring the necessity for comprehensive, coordinated care across specialties to optimize maternal and fetal outcomes.

## Introduction

Historically, women with congenital heart disease (CHD) were advised against pregnancy due to increased cardiovascular risks [1,2]. However, medical and surgical advancements now allow many women with complex CHD to live longer, healthier lives, leading to more women with complex cardiac histories choosing to pursue pregnancy. 

Hypoplastic left heart syndrome (HLHS) accounts for 2%-3% of all CHD. The 30-year survival rate is estimated to be greater than 80% [1,2]. HLHS is a prevalent congenital malformation, necessitating extensive surgical intervention and ongoing management throughout childhood. Following the Norwood, Glenn, and Fontan procedures, patients are left with single ventricle (Fontan) physiology, characterized by systemic circulation dependent on the right ventricle (RV) and passive venous return to the pulmonary arterial (PA) circulation. 

This manuscript reviews a complex case of a gravida 3, para 2 (G3P2) parturient with Fontan physiology, complicated by suspected placenta accreta spectrum (PAS) versus massive subchorionic thrombohematoma (MST), presenting with placental abruption at 32 + 2 weeks (32 weeks and 2 days) of gestation. This case underscores the complexities of managing pregnancy in patients with single ventricle physiology, the consideration of extracorporeal membrane oxygenation (ECMO) in high-risk scenarios, and the crucial need for coordinated care across multiple specialties to ensure optimal outcomes for both mother and fetus. 

To our knowledge, this is the first published case report describing the management of suspected PAS in a patient with Fontan physiology. This case is also notable for its unique multidisciplinary coordination and anesthetic management approach. The Health Insurance Portability and Accountability Act and written consent were obtained from the patient for the publication of this case report.

## Case presentation

A 29-year-old G3P2 woman with repaired HLHS presented for evaluation at 32 + 2 weeks of gestation. Her surgical history was positive for the classic staged palliation for HLHS, which includes the Norwood, bidirectional Glenn, and Fontan procedures, as well as an additional atrial septectomy. The patient had two prior cesarean sections performed at our institution. The first was a low transverse cesarean section, performed under epidural anesthesia, secondary to failure to progress during an induction of labor at 34 weeks. The second was a classical cesarean section, performed at 28 weeks due to preterm premature rupture of membranes (PPROM) and placental abruption, which was completed under combined spinal-epidural (CSE) anesthesia. 

The patient experienced no cardiopulmonary symptoms, such as shortness of breath, chest pain, lower extremity edema, or decreased exercise tolerance, prior to her first admission at 24 weeks of gestation during this third pregnancy. Her last cardiac catheterization occurred seven years earlier and indicated a well-functioning Fontan. 

**Figure 1:**
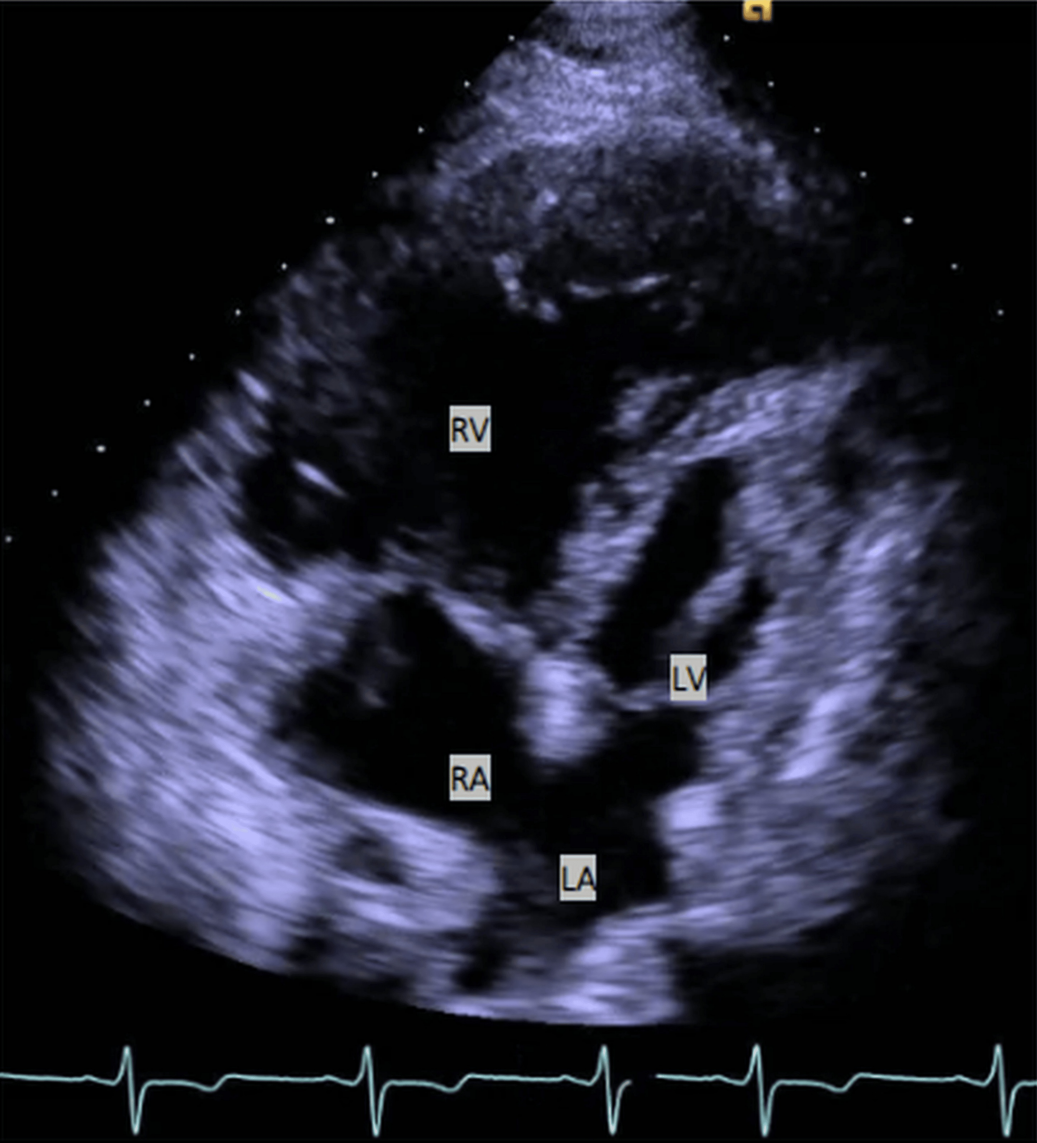
TTE showcasing an apical four-chamber view in a patient diagnosed with HLHS. The image highlights the classically hypertrophied systemic RV and the significantly underdeveloped LV. Additionally, the atria are visible, with a nearly absent septum dividing them - a result of a prior atrial septectomy. TTE, Transthoracic echocardiogram; HLHS, Hypoplastic left heart syndrome; RV, Right ventricle; LV, Left ventricle; RA, Right atrium; LA, Left atrium

**Figure 2:**
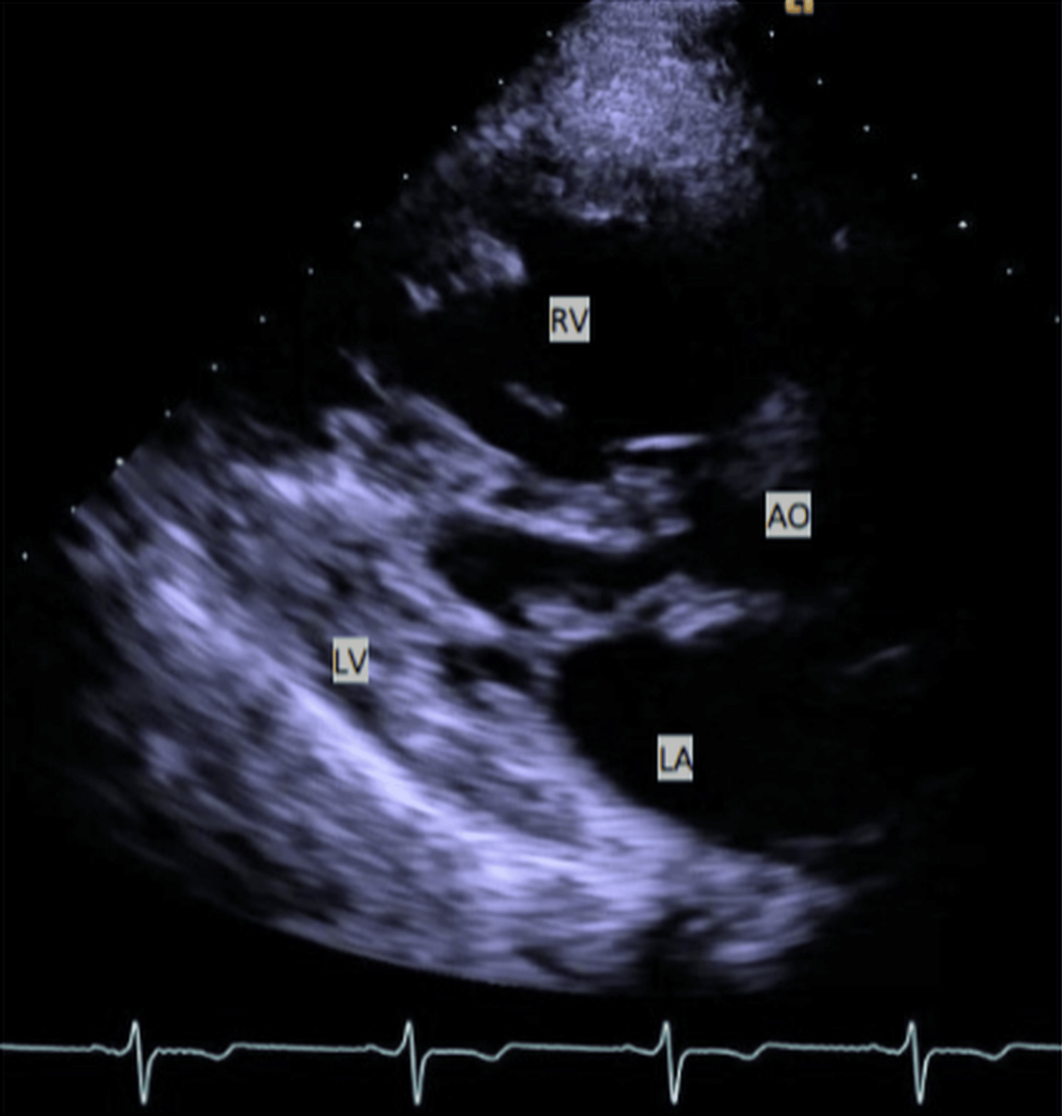
Parasternal long axis TTE view of the heart in a patient with HLHS. The image features the markedly hypoplastic LV and the systemic RV. TTE, Transthoracic echocardiogram; HLHS, Hypoplastic left heart syndrome; RV, Right ventricle; LV, Left ventricle; LA, Left atrium; AO, Aorta

A transthoracic echocardiogram (TTE) upon arrival revealed HLHS with an extracardiac Fontan, moderate hypertrophy, but normal systolic function of the systemic ventricle (native RV). The TTE findings indicated a mild gradient across the proximal descending thoracic aorta (peak velocity 1.8 m/s and pressure gradient 13 mmHg), consistent with the prior Norwood procedure (arch augmentation). The neoaortic valve (native pulmonary valve), arising from the systemic/dominant RV, showed no stenosis or regurgitation. The hypoplastic native aortic valve (annulus 1.3 cm), arising from the diminutive left ventricle, showed no stenosis (pressure gradient 6-7 mmHg) and mild regurgitation. The dominant/systemic RV was moderately hypertrophied with normal systolic function. There was trace to mild tricuspid valve regurgitation with no stenosis. The severely hypoplastic, diminutive left ventricle had low-normal systolic function (ejection fraction 50%-55%). The hypoplastic mitral valve (annulus measures 2.2-2.4 cm) showed mild mitral regurgitation. All of these findings were stable compared to her previous evaluations (Figures 1-2).

**Figure 3:**
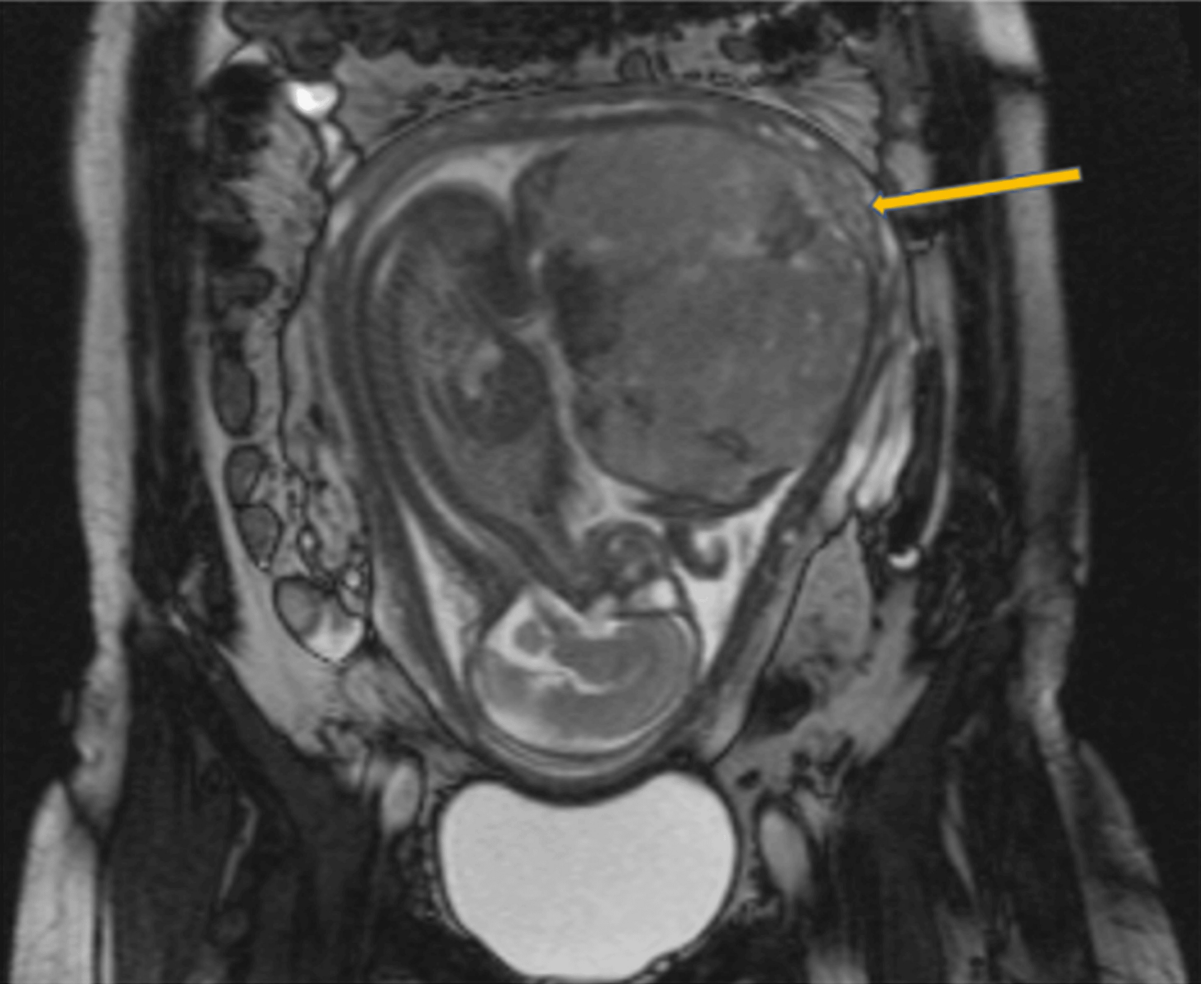
MRI of the pelvis performed without intravenous contrast at 32 weeks’ gestation. The image reveals a single intrauterine pregnancy in cephalic presentation, with the fetal spine oriented toward the maternal right. Notably, there are potential placental bands on the posterior left-lateral aspect of the placenta (demonstrated by the arrow), raising suspicion for PAS. MRI, Magnetic resonance imaging; PAS, Placenta accreta spectrum

Earlier in this pregnancy, the patient experienced a large subchorionic hemorrhage at 13 weeks of gestation, for which she was evaluated at our institution and discharged. She was later admitted to our hospital at 24 weeks due to PPROM, as mentioned above. She remained hospitalized for another four weeks and received latency antibiotics, which were given to reduce the risk of infection and improve neonatal outcome in the case of PPROM. During this time, ultrasound and magnetic resonance imaging (MRI) evaluations raised concerns for PAS versus MST. PAS encompasses conditions where the placenta abnormally adheres to the uterine wall, which can cause severe complications, like life-threatening hemorrhage during delivery, whereas MST is a rare pregnancy condition involving a significant maternal blood clot that separates the chorionic plate from the villous chorion, increasing the risk of placental abruption. In this specific case, the location of the placenta over the prior classical cesarean section scar and the MRI findings suggested PAS, while MST remained high on the differential, given this patient’s known first-trimester subchorionic hemorrhage, as mentioned above (Figure 3). Regardless of PAS or MST, the feto-maternal risk was high, with potential for severe consequences without intervention. 

A multidisciplinary team, including maternal-fetal medicine (MFM), cardiac and obstetric anesthesiology, cardiology, neonatology, and cardiothoracic surgery, developed a plan to manage both scheduled and emergent cesarean sections. The modified World Health Organization (WHO) classification was utilized for risk assessment. Fontan physiology is generally categorized as modified WHO Class III but may be Class IV when complications (e.g., ventricular dysfunction, cyanosis, Fontan-associated liver disease, and arrhythmias) are present [3]. Despite counseling, the patient chose to leave the hospital against medical advice due to childcare concerns, agreeing to return for weekly non-stress tests (NSTs) and ultrasound examinations until delivery. This patient was prescribed aspirin 81 mg daily for preeclampsia prophylaxis. She returned to the hospital at 32 + 2 weeks of gestation for vaginal bleeding attributed to placental abruption (Table 1). She reported bright red, bloody, mucoid, thick discharge approximately 3 inches in diameter, with abdominal pain rated 6/10 (cramps occurring every few minutes). 

**Table 1:** This table outlines key milestones and clinical observations throughout pregnancy. It is organized by gestational age, detailing associated events or symptoms, relevant clinical findings, and corresponding interventions or management strategies.

Gestational Age	Event/Symptom	Clinical Findings	Interventions/Management
13 weeks	Large subchorionic hemorrhage (SCH)	Ultrasound confirmed a large SCH	Patient evaluated and discharged; Outpatient follow-up
24 weeks	Preterm Premature Rupture of Membranes (PPROM)	Confirmed PPROM; High risk for infection and preterm delivery	Hospital admission; Latency antibiotics started
24-28 weeks	Inpatient monitoring due to PPROM	Magnetic resonance imaging (MRI) & ultrasound concerning for: PAS (Placenta Accreta Spectrum); MST (Massive Subchorionic Thrombohematoma)	Serial imaging; Differential diagnosis guided by placenta location and SCH history; Multidisciplinary team convened; Coordinated plan for cesarean delivery with maternal-fetal medicine (MFM), anesthesiology, cardiology, neonatology, and computed tomography (CT) surgery; Aspirin 81 mg daily for preeclampsia prophylaxis
28 weeks	Patient left against medical advice (AMA) due to childcare concerns	-	Agreed to return for weekly non-stress tests (NSTs) and ultrasounds
32 + 2 weeks	Vaginal bleeding with abdominal pain	Concern for placental abruption	Re-admission for evaluation and management of suspected placental abruption; Emergent cesarean section

The multidisciplinary team convened again and planned for a cesarean section via a vertical skin incision, a possible hysterectomy given suspected PAS, and a bilateral tubal ligation. The anesthesiology team included an obstetric anesthesiologist, a cardiac anesthesiologist, and a senior resident to manage intraoperative care. Additionally, MFM, gynecologic oncology, cardiothoracic surgery, and a perfusionist were present. The patient arrived in the operating room hemodynamically stable and without respiratory distress. The fetal heart rate tracing was normal. A radial arterial catheter was placed, followed by a left-sided internal jugular single-lumen Cordis introducer for both volume resuscitation and central venous pressure (CVP) monitoring. Finally, the anesthesia team inserted an 18-gauge angiocatheter into the right internal jugular (RIJ) vein for easy guidewire access and possible Seldinger upsizing for potential venous cannulation if venoarterial extracorporeal membrane oxygenation (VA-ECMO) was needed. The RIJ catheter was placed in addition to the femoral line to ensure adequate venous flow, given the patient’s Fontan physiology. 

Vasopressin (0.02 units/min) and norepinephrine (4 mcg/min) infusions were started in anticipation of CSE and were titrated to maintain mean arterial pressure (MAP) within 5%-10% of baseline (Table 2) and urine output greater than 0.5-1 mL/kg/hr. Vasopressin was chosen to maintain systemic vascular resistance (SVR) without increasing pulmonary vascular resistance (PVR), while norepinephrine was used to sustain SVR and enhance contractility. 

**Table 2:** Maternal hemodynamics key-points. NC, Nasal cannula

	Preoperative	Intraoperative	At Delivery	Postoperative
Blood pressure (mmHg)	129/60	163-103/85-51	136/59	114/64
Mean arterial pressure (mmHg)	87	108-70	84	81
Heart rate (bpm)	66	79-53	83	75
SpO_2_ (%)	97% (room air)	100-84% (2 L NC)	90% (2 L NC)	94% (2 L NC)

The CSE was performed in a seated position at the L2-L3 level, rather than the common L3-L4 location, based on favorable anatomical landmarks and MRI review. The anesthesia team proceeded with the administration of 1.2 mL of bupivacaine 0.75% with dextrose, fentanyl 15 mcg, and preservative-free morphine 100 µg. An epidural catheter was then threaded into the epidural space. Lidocaine 45 mg (without epinephrine) was selected as the test dose due to its hemodynamic neutrality. The test dose was negative following administration via the epidural catheter. Fentanyl was considered as an alternative test dose given the patient’s cardiac disease; however, plain lidocaine alone was determined to be adequate, and the test dose proved to be negative. 

Following CSE placement, the patient was positioned supine with left uterine displacement. The cardiac surgeon placed femoral arterial and venous sheaths for potential VA-ECMO cannulation. Following an uneventful delivery of the viable female infant (Apgar scores 5 and 6; venous cord gas: 7.30/25.0/43.0/-5), the MFM team noted that the placenta separated from the uterus normally, supporting a diagnosis of MST rather than PAS, and that hysterectomy was not indicated. A bilateral tubal ligation was then completed. The total quantified blood loss was 340 mL, intravenous fluids totaled 1000 mL, urine output was 300 mL, and no blood products were administered. The patient remained hemodynamically stable throughout the cesarean section and did not require VA-ECMO. 

Postoperatively, the patient was admitted to the ICU for close monitoring. The viable female infant was also monitored in the neonatal ICU, with an uneventful hospital course. The patient’s B-type natriuretic peptide was elevated (143 pg/mL on POD 0) but returned to baseline (39 pg/mL on POD 1) following one dose of intravenous furosemide. The sheaths (placed in preparation for VA-ECMO) were removed on POD 1. A postoperative echocardiogram was done on POD 1 without significant changes compared to the previous echocardiogram, with RV systolic function unchanged and no signs of heart failure. On POD 3, the patient was discharged home. At her initial postpartum follow-up visit, she continued to do well. 

## Discussion

Fontan physiology patients have undergone cardiac surgery in childhood for HLHS, resulting in a single functional ventricle that drives systemic circulation. Surgical palliation for HLHS involves three staged procedures: the Norwood, Glenn, and Fontan procedures. The Fontan procedure results in venous return directly from the vena cava to the PAs, as well as a single functional ventricle that is responsible for systemic cardiac output (CO) [4]. This results in multiple physiologic challenges, notably chronically elevated systemic venous pressures (SVPs), ventricular filling that is reliant mainly upon the pressure gradient between CVP and pulmonary artery pressure (PAP), as well as reduced CO given the weaker nature of the single ventricle compared to a normal left ventricle [5]. These challenges are aggravated in the parturient patient due to normal physiologic changes of pregnancy, which include increased blood volume and increased CO [6]. 

Unlike typical cardiac physiology, pulmonary flow in these patients is not driven by RV contraction, as the RV has assumed the responsibility of supplying systemic CO. Although not an entirely passive process, pulmonary blood flow (PBF), and thus preload to this systemic ventricle, mainly relies upon the pressure gradient between the CVP and PAP, the PVR, and the generation of negative intrathoracic pressure during spontaneous inspiration [7,8]. All of these factors are extremely sensitive to hemodynamic changes that occur in pregnancy. 

The normal physiological changes during a routine pregnancy pose significant challenges for women with Fontan physiology. By 20 weeks of gestation, blood volume and CO should increase by approximately 50% and 40%, respectively, while SVR and PVR decrease [6,9,10]. The necessary increase in CO may not be possible in Fontan patients, given the tentative nature of their single ventricle. The weaker myocardium may not improve stroke volume adequately, causing the heart rate to elevate in order to raise CO [6,7]. The increased blood volume of pregnancy raises CVP, thereby elevating filling pressures of the already preload-dependent, yet sensitive, single ventricle, which may cause congestive heart failure if this weaker systemic ventricle is not capable of producing adequate CO to offload itself. Luckily, the decreases in SVR in pregnancy help in offloading the single ventricle, while decreases in PVR help to promote forward flow to the single ventricle. Specific imaging evaluations (e.g., TTE) should consider the hemodynamic changes of pregnancy that peak between 28 and 32 weeks of gestation (typically 40%-50% above baseline) and plateau thereafter for parturients with CHD [6-8]. It is crucial to consider the timing of cardiac testing, particularly those performed before 24 weeks [9]. The hemodynamic changes that occur during delivery, particularly autotransfusion following fetal delivery, can significantly impact Fontan physiology. Autotransfusion, which involves the return of blood from the uterus to maternal circulation (approximately 500 mL), leads to a sudden increase in central blood volume, which may place considerable strain on the single ventricle, as mentioned above [9]. 

When managing a Fontan patient undergoing a cesarean section, a thorough evaluation of the patient's medical history, physical condition, and imaging is essential. Key components include assessing oxygen saturation and exercise capacity to determine baseline cardiovascular function. A detailed review of cardiac status, through echocardiography and cardiac catheterization reports, is necessary to understand the current state of the Fontan circulation. Additionally, a comprehensive surgical history should be obtained, noting any procedures related to Fontan-associated liver disease, as these can impact overall management and outcomes [6,10]. 

Anesthetic management

Effective communication and collaboration among the interdisciplinary team are critical for the successful management of patients with Fontan physiology. Key goals include maintaining PBF via sufficient preload, preventing elevation in PVR, preventing arrhythmias that can compromise CO, and attenuating increases in SVR, which can strain the systemic RV [9,11,12]. 

The preferred anesthetic plan for these patients typically involves neuraxial anesthesia. While there may be a decrease in preload, studies have shown good outcomes if the anesthetic is administered slowly and with close monitoring. Gradual preloading or co-loading of intravenous fluids, along with the initiation of vasopressors or inotropes prior to neuraxial anesthesia initiation, can be beneficial. Careful fluid management is essential to prevent volume overload and reduce strain on the systemic RV, while continuing to ensure adequate CVP to promote forward flow through the PA and into the single ventricle. Left uterine displacement should be implemented following CSE, given that patients with Fontan physiology may suffer dramatic drops in preload and deleterious elevations of afterload if aortocaval compression occurs. For labor and delivery, it is advisable to place the epidural early upon admission, in addition to invasive hemodynamic monitors. In emergent situations, a CSE approach is recommended, with the spinal component administered slowly, and general anesthesia may also be considered [13]. 

Given our experience with the patient’s two previous cesarean sections - one managed with epidural anesthesia and the other with CSE - we opted for the CSE technique again. Both times, the patient remained hemodynamically stable with vasopressin and norepinephrine infusions. However, the first delivery under epidural anesthesia was patchy and required epidural boluses and intravenous supplementation for adequate pain relief. A dural puncture epidural (DPE) was also considered. 

For general anesthesia in patients with Fontan physiology, careful monitoring and line placement are essential. A preinduction arterial catheter should be established, along with two peripheral intravenous lines (PIVs). Air filters on IV lines should be considered for patients with Fontan physiology - particularly those with a fenestrated repair - due to the mixing of blood within the right and left atria. This structural orientation increases the risk of air embolism, making air filters essential for preventing complications. Caution is needed, as some air filters may clog with propofol or blood. 

Ventilatory management should aim to optimize PBF. Spontaneous ventilation enhances venous return and promotes PBF. When mechanical ventilation is necessary, aim to limit peak inspiratory pressures, keep tidal volumes at 5-6 mL/kg, use lower respiratory rates, and limit positive end-expiratory pressure (PEEP) to avoid increasing intrathoracic pressure, which can impede venous return and reduce CO [7,12]. 

CVP and transesophageal echocardiography (TEE) monitoring are recommended if there are concerns about abnormal Fontan pressures, such as PVR exceeding 4 Wood units, mean pulmonary artery pressure (mPAP) greater than 15 mmHg, right ventricular systolic pressure (RVSP) greater than 50 mmHg, or worsening vital signs, such as reduced SpO_2_ and blood pressure. In such cases, it is advisable to call a cardiac anesthesiologist for support [6,13]. 

Volume resuscitation in postpartum hemorrhage poses unique challenges in patients with a single ventricle, as the heart relies almost exclusively on passive inflow from the pulmonary system, but is more vulnerable to fluid overload. The decision to prepare for ECMO in this asymptomatic, physically active patient centered around PAS and the risk of bleeding. While the average blood loss for a routine cesarean section is 700-1000 mL, the average blood loss for a cesarean hysterectomy for a patient with PAS is 2000-5000 mL. In these high-risk cases, it may be prudent to consider and prepare for ECMO support [8,13]. Uterine blood flow averages 500-800 mL/minute during routine pregnancy, and PAS-related hemorrhage could further reduce blood flow to the pulmonary vasculature and preload to the tenuous systemic ventricle, thus compromising cardiac function in a patient with Fontan physiology. In addition to both femoral arterial and venous sheaths, an RIJ angiocatheter was placed for potential venous cannulation, as there were concerns that femoral cannulation alone may not provide adequate venous drainage in this patient with abnormal central venous anatomy. Furthermore, VA-ECMO cannulation can take 30-60 minutes and can be especially challenging in a hypovolemic, hemorrhaging patient; therefore, planned cannulation was preferred over emergent. A Resuscitative Endovascular Balloon Occlusion of the Aorta (REBOA), a device used to reduce bleeding, was immediately available. A REBOA may allow for reduced bleeding and provide time for resuscitation, initiation of ECMO, or transportation to an ECMO-capable facility if needed. However, evidence for REBOA in PAS is evolving. 

Postpartum hemorrhage is a major concern, especially in patients with Fontan physiology, given their reliance upon adequate preload. The first-line uterotonic agent is oxytocin, followed by misoprostol. Methergine is typically avoided due to its potential to increase SVR and PVR, thereby increasing afterload on the weaker systemic ventricle in the case of increased SVR, and decreasing the pressure gradient between CVP and PAP in the case of increased PVR. Hemabate may cause bronchospasm and increase PVR, with similar pressure gradient effects. Though not uterotonic, tranexamic acid should be considered. In cases of confirmed PAS following fetal delivery, obstetricians may request that uterotonics not be given, as uterine contractions could lead to placental separation and subsequent bleeding. 

Postoperatively, a planned postpartum stay of two to three days, with echocardiographic monitoring, is recommended to assess and manage any cardiac changes. Individualized anticoagulation therapy should be tailored to the patient's needs, and vasopressor and inotropic support should be readily available to manage potential cardiovascular instability [13]. 

In our case, diagnostic imaging (e.g., MRI and ultrasound) failed to distinguish between MST and PAS. This may be due to similar imaging characteristics, such as abnormal placental attachment and blood collections on MRI, as well as resolution limitations preventing clear delineation of the subchorionic hemorrhage near the placenta. Fortunately, our team was adequately prepared in the case of PAS and massive hemorrhage. 

## Conclusions

This case underscores several important lessons in the management of complex obstetric patients with Fontan physiology. The diagnostic challenge of distinguishing PAS from MST has significant implications for surgical planning, risk assessment, and resource mobilization. In parturients with Fontan physiology undergoing high-risk surgery, early and proactive preparation for ECMO and REBOA can be considered to optimize outcomes. Multidisciplinary planning and a tailored anesthetic strategy are critical in parturients with CHD. As medical advances continue to extend the lifespan and reproductive possibilities for women with CHD, coordinated, multidisciplinary care remains essential to safely navigate the unique challenges of pregnancy in this population. 
